# Body composition and body mass index are independently associated with widespread pain and experimental pain sensitivity in older adults: a pilot investigation

**DOI:** 10.3389/fpain.2024.1386573

**Published:** 2024-07-01

**Authors:** Alisa J. Johnson, Jessica A. Peterson, Heather K. Vincent, Todd Manini, Yenisel Cruz-Almeida

**Affiliations:** ^1^ Department of Community Dentistry and Behavioral Science, Pain Research and Intervention Center of Excellence, College of Dentistry, University of Florida, Gainesville, FL, United States; ^2^ Department of Physical Medicine and Rehabilitation, College of Medicine, University of Florida, Gainesville, FL, United States; ^3^ Department of Health Outcomes and Biomedical Informatics, College of Medicine, University of Florida, Gainesville, FL, United States

**Keywords:** chronic musculoskeletal pain, fat mass, lean mass, body mass index, older adults

## Abstract

**Introduction:**

Chronic musculoskeletal (MSK) pain is prevalent in older adults and confers significant risk for loss of independence and low quality of life. While obesity is considered a risk factor for developing chronic MSK pain, both high and low body mass index (BMI) have been associated with greater pain reporting in older adults. Measures of body composition that distinguish between fat mass and lean mass may help to clarify the seemingly contradictory associations between BMI and MSK pain in this at-risk group.

**Methods:**

Twenty-four older adults (mean age: 78.08 ± 5.1 years) completed dual-energy x-ray absorptiometry (DEXA), and pain measures (Graded Chronic Pain Scale, number of anatomical pain sites, pressure pain threshold, mechanical temporal summation). Pearson correlations and multiple liner regression examined associations between body mass index (BMI), body composition indices, and pain.

**Results:**

Significant positive associations were found between number of pain sites and BMI (*b = *0.37) and total fat mass (*b *= 0.42), accounting for age and sex. Total body lean mass was associated with pressure pain sensitivity (*b *= 0.65), suggesting greater lean mass is associated with less mechanical pain sensitivity.

**Discussion:**

The results from this exploratory pilot study indicate lean mass may provide additional resilience to maladaptive changes in pain processing in older adults, and highlights the importance of distinguishing body composition indices from overall body mass index to better understand the complex relationship between obesity and MSK pain in older adults.

## 1 Introduction

Chronic musculoskeletal (MSK) pain is a prevalent and disabling condition in older adults, negatively affecting quality of life and physical independence (1). Obesity, as defined by a body mass index (BMI) ≥30 kg/m^2^, has been identified as a risk factor for developing MSK pain (2). However, the relationships between BMI and MSK pain are complex. Cross sectional data show that both high or low BMI values are associated with an increased risk for general chronic MSK pain (3).Furthermore, sensory pain processing may be altered in obese individuals which may place them at a higher risk for developing chronic MSK pain compared to individuals with healthy weight (4).

Body composition measures may improve the clarity of the relationships between body weight and bodily pain, and pain sensitivity. The current state of the evidence is mixed. There is evidence that suggests fat mass, but not lean tissue mass, is related to low back pain severity (5). In knee osteoarthritis (OA), both BMI and intramuscular fat infiltration were associated with greater pain and disability burden cross-sectionally and longitudinally (6, 7). However, another study in knee OA suggests intermuscular fat infiltration was not associated with pain severity (8). Aging itself is associated with increased total body fat mass and decreased lean mass, independent of BMI (9), and higher body fat has been shown to increase the risk for developing cardiovascular disease, type 2 diabetes, and sarcopenia (10), conditions which are often comorbid with chronic MSK pain (10–12). Therefore, examining body composition may help to clarify tissue types driving the development and persistence of pain and pain tolerance among older adults with different BMI values.

While BMI is a commonly-used easy and inexpensive way to categorize weight status across the age spectrum, it cannot discriminate proportions of lean mass and fat mass for a given body weight. While it is recognized that high BMI is likely associated with elevated body fat (with normal or low muscle mass), it is possible to have a high BMI related to high muscle mass. Conversely, low BMI is typically associated with low fat and low muscle mass, but can also be associated with high proportions of fat and relatively low muscle mass. Given that BMI is a non-specific body composition measure, the role of increased fat mass, or decreased muscle mass in chronic MSK pain remains unclear. One method to dichotomize tissue type (fat mass vs. lean mass) is the use of dual-energy x-ray absorptiometry (DEXA), which may help to further clarify the complex relationship between body mass, body composition, and chronic pain in older adults (13). Therefore, the purpose of this exploratory pilot study was to examine associations between body composition variables and clinical and experimental pain sensitivity measures, independent of BMI, in older adults (age = 70 + years) at risk for chronic pain. Based on prior literature (13), we hypothesized that greater total body fat mass would be positively associated with clinical and experimental pain sensitivity, and that greater total body lean mass would be negatively associated with clinical and experimental pain sensitivity.

## 2 Materials and methods

### 2.1 Study population

A total of 24 community-dwelling older adults (age range: 77.86 ± 5.03 years) were recruited through the University of Florida (UF) as part of a local pilot study ancillary to a larger inflammation and aging study. Full study details have been previously described (14). Briefly, individuals ages 70 years and older were screened for eligibility, over the course of four contacts, which included: (1) phone screen (e.g., age, self-reported walking difficulty), (2) usual walking speed (<1 m/sec on 4 m walk test), (3) able to complete 400 m walk test within 15 min, and (4) blood level IL-6 between 2.5 pg/ml and 30 pg/ml. Individuals were excluded if: (1) unable to provide informed consent, (2) lived in nursing home, (3) self-reported inability to walk one block, (4) significant cognitive impairment (Mini Mental State Exam <24), (5) unable to communicate due to speech or hearing loss, (6) neurological condition (e.g., stroke with paralysis, neuropathy, Parkinson's, multiple sclerosis), (7) severe rheumatologic, autoimmune/inflammatory, or orthopedic disease (e.g., rheumatoid arthritis, lupus, Crohn's, HIV, awaiting joint replacement), (8) terminal illness, (9) pulmonary disease, (10) other significant comorbidities that would impair participation, and (11) lives outside study area or planning to move in the next year.

### 2.2 Study design

In this cross-sectional ancillary pilot study, participants completed additional study visits at baseline, prior to the initiation of the intervention. These visits included measures of clinical pain, experimental pain, and body composition variables. Participants also reported demographic information (e.g., age, sex, education, income, and race), and were screened to determine eligibility for DEXA scanning (e.g., no thyroid, heart, lung, or liver scans in the past year, no upper gastrointestinal series or barium enema in the past seven days, no metal implants in bones). The study was approved by the UF Institutional Review Board (IRB, study #201600433) and participants completed written informed consent prior to study procedures.

### 2.3 Measures

#### 2.3.1 Clinical pain sensitivity

A Pain History Questionnaire asked participants to indicate body sites from at which they experienced MSK pain during the past three months (head, neck/shoulders, lower back, extremities). The total number of pain sites was calculated and used in the analysis (15). The Graded Chronic Pain Scale (GCPS) (16), assessed characteristic pain intensity and pain-related disability over the past six months. Participants were asked to indicate on a 0 (no pain/no interference) to 10 (pain as bad as could be/unable to carry on activities) numerical rating scale (NRS) current, worst, and average pain over the past six months, as well as how much pain had interfered with daily activities, ability to participate in social and recreational activities, and ability to work over the past six months. Scores were averaged and multiplied by 10 to compute characteristic pain intensity and pain-related disability scores, respectively.

#### 2.3.2 Experimental pain sensitivity

Experimental pain sensitivity was assessed using a standardized protocol. A digital handheld algometer (AlgoMed, Medoc, Ramat Yishai, Israel) was used to apply pressure at a constant rate of 30 kPa/sec up to a maximum pressure level of 1000kPa. Participants were instructed to press a button they were holding when the pressure sensation first became painful [i.e., Pressure Pain Threshold (PPT)]. The trial was repeated three times on the belly of the trapezius muscle and then averaged. Higher scores indicate less pain sensitivity. Mechanical punctate temporal summation (TS) was assessed using a 300 g von Frey filament applied to the thenar eminence (i.e., hand) and the first metatarsal (i.e., foot). Participants rated their pain on a 0 (no pain) to 100 (worst pain imaginable) NRS after a single, and then a series of 10, stimuli. TS was calculated as the difference between the single pain rating, and the pain rating following the 10 stimuli by testing site. Higher values indicate greater TS.

#### 2.3.3 Body composition

Participants completed DEXA scans to determine total body fat and total body lean mass using a standardized procedure. Body mass index (BMI) was calculated as kg/m^2^ using height and weight measurements taken prior to the DEXA scan.

### 2.4 Statistical analysis

Data were assessed for normality and outliers prior to analysis. Descriptive statistics were calculated as means and standard deviations for continuous variables and counts and percentages for categorical variables. We examined scatterplots and bivariate Pearson correlations to examine linearity among variables. Multiple linear regression determined associations between BMI (Model 1) and body composition measures (i.e., total fat mass, total lean mass; Model 2), with clinical pain sensitivity (i.e., total number of pain sites, GCPS pain intensity, and GCPS pain interference), and experimental pain sensitivity (i.e., pressure pain threshold and temporal summation). Age and sex were entered as covariates given their known associations with BMI and pain (17). A power analysis was conducted in G*Power 3.1.9.7 software (18), and indicated for the current sample size (*n* = 24), we were powered (1-β=0.80) to detect a large effect size (*ρ=0.*50), with two-tailed α=0.05. Data analyses were performed using IBM SPSS v.27 software.

## 3 Results

### 3.1 Sample

Twenty-four participants from the ENRGISE-Ancillary pilot study completed measures of interest and are included in this analysis. Table 1 presents demographic and clinical characteristics. The majority of participants in the current sample were male (75%), and Caucasian/White (87.5%). The mean age of the sample was 78 years (SD = 5.1; range = 72–91 years). Most of the participants reported income less than the state-median (73.7%). On average, participants reported experiencing pain on most days over the past three months at two anatomical sites, with pain more commonly reported at lower body sites (i.e., foot, knee, and/or hip). Bivariate correlations between BMI and total body fat mass (*r *= 0.79, *p* < 0.001), and BMI and total body lean mass (*r *= 0.63, *p* = 0.001) were statistically significant with total body fat explaining 62% of the variance in BMI, and lean body mass accounting for 40% of the variance in BMI. However, the correlation between total body fat mass and total body lean mass was not statistically significant (*r *= 0.26, *p* = 0.21).

**Table 1:** Sample characteristics.

Variable, *M ± SD* or *N* (%)	Total sample (*n* = 24)	Male (*n* = 18)	Female (*n* = 6)
Age, years	78.08 ± 5.1	77.8 ± 4.7	78.8 ± 6.7
Race			
AA/Black	3 (12.5)	1 (5.6)	2 (33.3)
Caucasian/White	21 (87.5)	17 (94.4)	4 (66.7)
Education			
No college	11 (45.8)	9 (50.0)	2 (33.3)
College	13 (54.2)	9 (50.0)	4 (66.7)
Income			
≤$55k	14 (73.7)	10 (71.4)	4 (80.0)
>$55k	5 (26.3)	4 (28.6)	1 (20.0)
Body mass index (BMI)	30.63 ± 4.7	31.1 ± 4.9	29.3 ± 4.3
Total fat mass (kg)	32.90 ± 9.7	32.2 ± 9.5	35.0 ± 10.8
Total lean mass (kg)	55. 66 ± 10.3	59.4 ± 8.6	44.4 ± 6.0
Total pain sites, *Mdn (IQR)*	2.0 (3.0)	1.5 (3.25)	3.5 (1.75)
GCPS pain intensity	36.81 ± 21.4	35.7 ± 20.4	40.0 ± 25.6
GCPS pain interference	27.68 ± 25.0	23.5 ± 23.1	39.4 ± 28.7
PPT trapezius (kPa)	760.67 ± 267.9	809.8 ± 275.8	584.0 ± 146.1
TS hand	13.78 ± 21.9	4.8 ± 7.4	40.8 ± 29.1
TS foot	13.78 ± 20.4	7.2 ± 10.7	36.0 ± 30.4

AA, African American; GCPS, graded chronic pain scale; PPT, pressure pain threshold, TS, temporal summation = difference in pain rating (0–100 scale); Mdn, median; IQR, interquartile range; PPT, pressure pain threshold (higher values indicate less pain sensitivity); TS, temporal summation (higher values indicate greater pain sensitivity).

### 3.2 Associations between body composition and clinical pain

Multiple linear regression demonstrated that BMI was positively associated with total number of pain sites, adjusting for age and sex, [*b *= 0.37, 95% CI: (0.01,0.27), *R*^2 ^= 0.54], suggesting greater BMI is associated with higher total number of painful body sites. In a separate model, body fat mass was positively associated with total number of pain sites, accounting for age, sex, and total lean body mass [*b *= 0.42, 95% CI:(0.01,.015), *R*^2 ^= 0.55]. There were no statistically significant associations between body composition indices and GCPS pain intensity or GCPS pain interference, Table 2.

**Table 2:** Multiple linear regression models: clinical pain sensitivity.

Variable	*b*	*p*	95% CI	R^2^
Total pain sites (model 1)				0.537
Age	−0.271	0.103	−0.22, 0.27	
Sex	**0**.**611**	**<0**.**001**	**1.22, 3.92**	
BMI	**0**.**366**	**0**.**034**	**0.12, 0.274**	
Total pain sites (model 2)				0.552
Age	**−0**.**389**	**0**.**043**	**−0.28, −0.01**	
Sex	0.366	0.125	−0.47, 3.55	
Total fat mass	**0**.**417**	**0**.**027**	**0.01, 0.15**	
Total lean mass	−0.225	0.394	−0.14, 0.06	
GCPS pain intensity (model 1)				0.042
Age	0.190	0.440	−1.30, 2.87	
Sex	0.104	0.656	−17.95, 27.85	** **
BMI	**0**.**121**	0.629	−1.85, 2.99	** **
GCPS pain intensity (model 2)				0.185
Age	−0.027	0.917	−2.29, 2.08	** **
Sex	−0.329	0.311	−47.24, 15.91	** **
Total fat mass	0.323	0.192	−0.41, 1.88	** **
Total lean mass	−0.606	0.108	−2.75, 0.30	
GCPS pain interference (model 1)				0.084
Age	0.000	0.998	−2.39, 2.39	
Sex	0.297	0.203	−9.71, 42.79	
BMI	0.052	0.832	−2.49, 3.06	
GCPS pain interference (model 2)				0.164
Age	−0.157	0.546	−3.35, 1.83	
Sex	−0.021	0.949	−38.64, 36.32	
Total fat mass	0.213	0.390	−0.79, 1.92	
Total lean mass	−0.460	0.222	−2.90, 0.72	

GCPS, graded chronic pain scale; BMI, body mass index; *b*, standardized coefficient. Bolded values indicate statistical significance at 0.05 level.

### 3.3 Associations between body composition and experimental pain

The results from multiple linear regression showed that total body lean mass was positively associated with pressure pain threshold at the trapezius adjusted for age, sex, and total body fat mass [*b *= 0.65, 95% CI: (0.80, 34.3), *R*^2 ^= 0.38], indicating greater lean mass was associated with less pain sensitivity to pressure. There were no other significant associations between body mass index or body composition variables and experimental pain indices. The model summaries are provided in Table 3.

**Table 3:** Multiple linear regression models: experimental pain sensitivity.

Variable	*b*	*p*	95% CI	R^2^
PPT trapezius (model 1)				0.258
Age	−0.220	0.312	−40.04, 13.49	
Sex	−0.350	0.103	−493.30, 49.33	
BMI	0.228	0.296	−11.98, 37.23	
PPT trapezius (model 2)				0.380
Age	−0.066	0.764	−31.45, 23.49	
Sex	0.050	0.858	−336.45, 400.26	
Total fat mass	−0.229	0.292	−18.24, 5.81	
Total lean mass	**0**.**646**	**0**.**041**	**0.80, 34.35**	** **
TS hand (model 1)				0.594
Age	0.180	0.239	−0.551, 2.09	
Sex	**0**.**692**	**<0**.**001**	**19.33, 49.13**	
BMI	−0.133	0.386	−2.06, 0.83	
TS hand (model 2)				0.620
Age	0.276	0.111	−0.30, 2.65	
Sex	**0**.**891**	**<0**.**001**	**22.29, 65.81**	** **
Total fat mass	−0.220	0.187	−1.26, 0.26	
Total lean mass	0.246	0.312	−0.53, 1.57	
TS foot (model 1)				0.436
Age	0.183	0.343	−0.89, 2.42	
Sex	**0**.**533**	**0**.**009**	**7.01, 43.70**	** **
BMI	−0.157	0.415	−2.29, 0.99	** **
TS foot (model 2)				0.464
Age	.0135	0.518	−1.25, 2.38	
Sex	0.479	0.085	−3.49, 49.13	** **
Total fat mass	−0.161	0.433	−1.21, 0.54	
Total lean mass	−0.160	0.599	−1.50, 0.89	

BMI, body mass index; PPT, pressure pain threshold; TS, temporal summation; *b*, standardized coefficient. Bolded values indicate statistical significance at 0.05 level.

## 4 Discussion

Findings from this exploratory pilot study suggest that total body fat mass, total body lean mass, and BMI, are differentially associated with clinical and experimental pain sensitivity measures in older adults. Our hypothesis was partially supported such that: (1) total body fat mass and body mass index (BMI) were each positively associated with the total number of self-reported anatomical pain sites, and (2) total lean mass was associated with less pressure pain sensitivity, supporting prior evidence of the relationship between body composition and pain (13). Surprisingly, BMI and body composition measures were not significantly associated with other clinical or experimental pain outcomes.

While obesity as a health condition has been consistently identified as a risk factor for chronic MSK pain, previous research has relied predominantly on BMI to define obesity, with less consideration for tissue type (19). Thus, these findings help to address critical knowledge gaps regarding fat and lean body mass contributions to the development and persistence of chronic MSK pain, especially among older adults. BMI cannot explain differences in muscle fat infiltration, intramuscular compounds (metabolites) and production of proinflammatory cytokines and chemokines that each may facilitate pain (20). In the current study, we found that greater lean mass was associated with less widespread pain, supporting the protective role of lean tissue mass in older adults. This is aligned with another study reporting sarcopenic obesity (i.e., high body fat percentage accompanied by low skeletal mass and muscle function) (21), was associated with an increased risk for painful knee OAs, compared to either obesity or sarcopenia alone (22). Metabolic factors may underlie the relationship between obesity and chronic MSK, including increased local oxidative stress and pro-inflammatory processes (23, 24). Given that the current sample was prevalently obese (mean BMI = 30.6), it is possible that participants had fat infiltration into skeletal muscle (myosteatosis) (25), higher systemic inflammation, which may be compounded by a diet that favors inflammation (26). Age-related accrual of adipose tissue can increase systemic inflammation (27–29), and the pro-inflammatory signaling from adipose tissue (30), may activate and sensitize nociceptors leading to heightened pain sensitivity (31, 32). Our finding that greater total body fat mass was related to enhanced pressure pain sensitivity is consistent with this idea, as pressure pain sensitivity at the trapezius is an indicator of pain sensitization (33, 34). Unfortunately, we did not measure systemic inflammation in the present sample, but future studies could explore this hypothesis.

In this study, we found that lean mass was inversely associated with pressure pain threshold at the trapezius, while temporal summation measurements were only associated with sex, such that females had significantly higher levels of temporal summation. Temporal summation is a dynamic quantitative sensory testing method that invokes neural mechanisms related to pain facilitation (35), and has been associated with worse clinical pain reports (36, 37). While other studies in adolescents found arm-specific lean mass was associated with conditioned pain modulation (CPM) and temporal summation (38, 39), in adults (aged 18–45 years), CPM responses were not associated with lean or fat mass (40). These contrasting findings may be due to age differences in pain modulatory processes. Given that CPM is a proxy measure for endogenous pain inhibition, this assessment may reflect age-related changes in somatosensory function and skeletal muscle phenotypes. Our findings also suggest that in older adults, sex-related pain differences persist independent of BMI or body composition. Interestingly, Peterson et al. (41) found that lower muscle mass rather than higher fat mass could be a contributing factor to a heightened mechanical pain sensitivity in the same group of adults, which is in line with the current study findings that greater total lean mass was associated with less mechanical pain sensitivity. No study to date has tested this association in older adults, specifically among those experiencing chronic MSK pain. Our current findings provide preliminary evidence that total body lean mass and pain sensitivity may be related, and preservation of lean mass may protect against age-related changes in pain processing (42). As quantitative sensory testing (i.e., mechanical) assesses function across the entire sensory neural axis, dysfunction in peripheral nerves including those located in peripheral lean tissue, may contribute to alterations in somatosensory function that occur as we age (43, 44). Furthermore, age-related loss of muscle fibers and motor units commensurate with defective regeneration playing a role in peripheral nervous system function, both sensory and motor, specific to lean tissue amount.

### 4.1 Limitations and future considerations

This study has several considerations to note; firstly, while the study was adequately powered, the sample size was small. Second, this was a secondary data analysis and other measures which may have been relevant to our investigation were not collected (e.g., global strength, measures of thermal pain sensitivity, systemic inflammation, physical activity type and amount, dietary intake of antioxidants, comorbidity, depression), and the inclusion/exclusion criteria of the parent study may have reduced generalizability of the findings. Further research examining the role of lean mass in chronic MSK pain outcomes and pain sensitivity in older adults is warranted, including studies assessing these relationships in larger samples with more racial diversity, and including potentially-confounding factors such as comorbidity and depressive status. Moreover, the pain duration and anatomical location may impact physical activity levels and subsequent muscle composition due to increased inactivity. Sarcopenia represents one of the most prevalent causes of functional decline and loss of independence in older adults; the systematic investigation of sarcopenia in obese and underweight older adults may further elucidate the complex relationship between chronic MSK pain and body composition in this population. In addition, there is growing recognition of the role of muscle composition as it relates to muscle function, not just muscle size, and that muscle function is likely a more salient contributing component to long-term functional outcomes in persons with chronic MSK pain. Longitudinal tracking of body composition change, fat deposition pattern (central, gynoid) changes, muscle structure and function, pain site location and severity, and pain sensitivity measures would provide the optimal opportunity to understand these relationships in older adults. Future studies which included assessments of inflammatory markers and dietary patterns would provide novel and valuable information in understanding the complex relationships between BMI and MSK pain.

### 4.2 Conclusions

Our findings show that total lean body mass was inversely associated with clinical pain ratings and temporal summation, and total body fat mass is directly associated with mechanical pain sensitivity. Body composition, rather than BMI, may provide a more nuanced understanding of the factors contributing to increased chronic MSK pain risks among obese and underweight older adults, and lead to the development of improved preventative and intervention strategies. It will be important to continue this line of research including investigating the potential utility of other clinically available measures of body composition (e.g., bioelectrical impedance analysis), to fully appreciate the factors underlying increased MSK pain.

## Data Availability

The raw data supporting the conclusions of this article will be made available by the authors, without undue reservation.
